# Supplementary material for: Are carbon emissions trading and green financial instruments synergistic? -Comprehensive quantitative research based on content analysis
Source: PLoS One. 2024 Mar 7;19(3):e0298601. doi: 10.1371/journal.pone.0298601 (PMC10919601; doi:10.1371/journal.pone.0298601)
Supplement: S1 Data — (ZIP) [file pone.0298601.s001.zip › Data/地级市Z_F工作报告.zip!/╦─┤¿/╣π░▓╩╨/└┤╫╘╣1⁄2╓┌║┼í╛┬φ┐╦╩2╛▌═°í┐_2016.docx]

各位代表：

　我代表市人民政府，向大会作政府工作报告，请各位代表审查，并请市政协委员提出意见。

2015年工作及“十二五”简要回顾

　过去一年，在市委坚强领导下，在市人大、市政协监督支持下，我们坚持新常态有新作为，努力抢抓重大机遇，积极应对多重挑战，深入实施“三大发展战略”，统筹稳增长、调结构、促改革、惠民生、防风险，较好完成了市四届人大六次会议确定的目标任务。全年实现地区生产总值1005.6亿元、增长10.6%，增速居全省第3位；完成全社会固定资产投资1132亿元、增长23.1%，增速居全省第2位；实现地方一般公共预算收入56.7亿元、增长22.8%，增速居全省第2位；城乡居民人均可支配收入分别增长8.1%、10.3%。

　一年来，主要做了以下工作。

全力以赴稳定经济增长。针对宏观经济环境偏紧，经济下行压力持续加大，我们及时研判经济走势，先后制定出台一系列稳增长政策措施，努力破解企业、行业和实体经济发展难题，推动了经济持续较快增长。突出抓好项目投资。争取到中省重大项目1323个、到位补助资金54.2亿元；广安电子信息研发生产销售一体项目、年产10万吨工业磷酸生产线等项目开工建设，广安聚苯硫醚树脂生产、华润雪花啤酒厂搬迁扩能等项目加快建设，仁安药业抗生素及原料药生产线、诚信化工二期等项目建成营运，529个投资1000万元以上重大项目累计完成投资700亿元、增长21.6%。投资对经济增长的贡献率达60%以上。大力拓市场促消费。扩大健康、养老、电商等新兴消费和服务供给，积极参加全川市场拓展“三大活动”，不断完善城乡流通体系，培育大型卖场3个、骨干专业市场6家，社会消费品零售总额增长12.7%。外贸进出口总额达10.5亿美元；实际利用外资4287万美元。狠抓工业稳定增长。出台并兑现落实工业经济稳定增长20条措施，积极开展“入园进企帮扶”活动，帮助企业解决融资、用电、用气、用工等具体困难，推动工业经济逆势上行，实现工业总产值1475亿元、增长12.8%，增加值增长12.4%、居全省第3位。促进房地产业健康发展。落实房地产市场新政，调整公积金提取办法，完成房地产开发投资184.3亿元，商品房竣工面积113万平方米，销售面积299.5万平方米。发挥财政金融支持实体经济作用。设立5000万元应急转贷资金，建立3亿元扶持实体经济发展专项资金，投放2.15亿元支持28家实体企业。城市地下综合管廊建设工程等44个项目纳入国家专项建设基金项目，获得国开行、农发行专项建设基金注入资本金18.5亿元。全年新增贷款67.2亿元、增长13.1%。

着力推动产业转型升级。加快工业转型发展。依托创新培育工业经济发展新优势，重点推进生物医药、电子信息、节能环保与新材料、高新技术、新型住宅制造、精细化工、高端装备制造等战略性新兴产业和高端产业发展，实现产值829亿元、增长36.5%。推动传统优势产业改造升级，有序消化过剩产能，淘汰落后产能，完成工业技改投资156亿元。深入实施大企业大集团培育计划，银钢凸轮在“新三板”成功挂牌，领创电子、金侑达电业等24户企业进入成都川藏股权交易中心展示；加快中小微企业培育发展，新增小微企业1280户。工业布局持续优化，“一园一主业”基本成型，园区配套功能进一步完善，园区工业产值、利税分别占全市规上工业企业的86.7%和85%。推动现代服务业改革发展。扎实推进省级服务业综合改革试点市和现代物流业试点示范市建设，六大新兴先导型服务业加快发展，培育省级现代服务业集聚区2个，实现服务业增加值322.1亿元、增长12%，增幅居全省第1位。电子商务加快发展，岳池县、广安区分别纳入国家级、省级电子商务进农村示范县，武胜县电子商务产业园纳入全省重点电子商务集聚区，新引进培育电商企业20户，实现电子商务交易额80亿元、网络零售额12亿元。现代物流业蓬勃发展，引进远成物流等知名物流企业3家，培育A级物流企业2家，完成物流业投资229亿元、增长25.2%。旅游经济不断壮大，重点旅游项目完成投资65亿元、增长39%；实现旅游收入245.5亿元、增长26.2%。保险服务业健康发展，实现保费收入33亿元，理赔金额11亿元。推动农业发展方式加快转变。加快国家现代农业示范区和整体推进现代农业产业基地试点市建设，深入推进产村相融＂111＂示范工程，新（改）建现代农业产业基地25万亩，新（改、扩）建畜禽标准化规模养殖场52个。稳定粮食生产能力，实现粮食总产189.4万吨。推进现代农业标准化生产、产业化经营，培育新型农业经营主体6213个，加强邓小平故里优质农产品“华蓥山”公用品牌建设，广安区、岳池县成功创建省级地理标志产品保护示范区，申报认证“三品一标”农产品56个，广安农产品影响力和市场竞争力不断提升。

全面深化改革开放创新。进一步细化重点领域改革时间表、路线图，新推出32项改革方案。推进简政放权改革，公布市本级行政许可项目目录230项，全面取消非行政许可审批事项；推进公共资源交易机制改革，实现电子交易平台流程再造；商事制度改革有序推进，“三证合一、一照一码”深入实施，全市新登记个体工商户17072户、私营企业2757户，分别增长17.4%、28.3%。扎实推进金融改革，华蓥农商行、邻水农商行召开创立大会，广安思源农商行、成都农商行开业营运。稳步推进农村改革，全面完成农村土地确权登记颁证工作；六区市县全域纳入全省农村产权抵押融资试点范围，武胜县被列为全省农村集体资产股份合作制改革试点县。财政支农资金形成资产股权量化改革试点工作有序开展。深化投融资体制改革，推广运用政府和社会资本合作模式，启动实施PPP项目5个、总投资81.11亿元。稳步推进财税体制改革，出台广安市财税体制改革总体方案。国资国企改革稳步实施。新组建广安金财投融资集团、四川经准检验检测集团。研究出台规范国有企业投融资及国有产权处置等管理办法，促进了国有企业规范健康发展。公务用车、不动产统一登记制度改革有序推进。大力促进创新创业。研究出台全面推进大众创业、万众创新实施意见和加快高新技术产业发展的实施意见，制定实施“互联网+”行动计划，创新创业活力加速释放，新创建国家高新技术企业9家，新增科技型中小微企业80家，新培育市级产业技术研究院5所，建成省级创业示范基地（创业孵化园）3个、大学生创新创业园6个。发明专利申请量达1050件，转化重大科技成果25个。全方位推进开放合作。主动融入“一带一路”、长江经济带发展战略，加强川渝合作示范区、国家西部承接产业转移示范区建设，深化与重庆、深圳、天津滨海新区务实合作，在基础设施、产业发展、公共服务、人才交流等领域合作取得新的进展。与北京中关村、中国电子信息产业研究院、清华大学机械工程学院建立战略合作关系。积极参加“中外知名企业四川行”、“香港四川活动周”等重大投资促进活动，成功引进项目683个、招商项目实现固定资产投资503.6亿元。

统筹推进城乡区域发展。有序推进以人为核心的新型城镇化。广安市城市总体规划（2013-2030）获省政府批复。奎阁、枣山、协兴、官盛、前锋五大片区联动发展，骨架道路基本形成；滨江路防洪综合改造、高岩人行天桥、环溪一路综合改造等主城区市政配套工程有序实施；加快智慧城市建设，开展“双违”治理，城市管理水平不断提升。推进特色集镇及幸福美丽新村建设，完成47个特色集镇总体规划及43个控制性详细规划编制，投入资金9.6亿元推进“七个一”工程建设，建成幸福美丽新村221个、新农村聚居点388个。新增城镇面积18平方公里，城镇化率提高1.8个百分点。进一步完善城乡基础设施。着力完善城乡交通体系，官盛大桥、枣山公路客运枢纽站等项目开工建设；广华快速通道、G350线武胜过境段、G210邻水县城西环线加快建设；巴广渝、遂广高速公路广安段以及广岳大道建成通车；G42邻水西新建、华蓥西提档改造竣工通车；广安港新东门作业区一期、富流滩二线船闸、渠江航道整治工程基本完成；开通广安至前锋城市公交；建设农村公路872公里。扎实推进农田水利建设，向阳桥、龙滩水库开工建设，猫儿沟、回龙寺、应家沟水库建设加快推进，奎阁、罗渡、海棠溪、明月等堤防工程加快实施，完成病险水库整治17座，新解决36万农村人口饮水安全问题，建成农建示范区12万亩、高标准基本农田20.5万亩，开展国土整理25万亩。大力推进县域经济发展和园区建设。深入贯彻落实加快县域经济改革发展政策措施，支持广安区现代服务业、前锋轻纺加工、华蓥电子信息、岳池生物医药、武胜节能环保、邻水装备制造等主导产业做大做强，县域经济实力不断壮大。理顺市级园区管理体制机制，园区发展活力不断增强。

大力发展民生社会事业。民生支出达158亿元，占地方一般公共预算支出比重70.2%。全面或超额完成省上110项民生工程和民生实事任务，实施了8大类24项市自办民生工程。完善就业创业政策，新增就业人员5.59万人，实现再就业1.4万人，登记失业率控制在3.29%以内。开展“百万安居工程建设行动”，开工建设城镇保障性住房和改造危旧房棚户区29072户，改造农村危房8533户。整治地灾隐患54处、避险搬迁835户。新建村卫生室200个，实施农村妇女“两癌”免费检查22000例，对300名白内障患者免费实施复明手术。为15万残疾人提供个性化服务。大力实施精准扶贫精准脱贫。出台脱贫攻坚政策措施，制定10个专项扶贫方案，完成820个贫困村扶贫规划编制，深入推进“五大工程”，扎实开展驻村帮扶，强化社会参与扶贫，筹集扶贫资金3.31亿元，帮助5.77万名贫困人口实现脱贫。持续加强社会保障。打通城乡居民养老保险与企业职工养老保险制度转移衔接通道，17.5万名灵活就业人员参加企业职工基本养老保险，城乡居民养老保险覆盖129.63万人，机关事业单位养老保险制度改革有序推进；与川渝两地137家定点医院实现异地就医即时结算；30.5万困难群众应保尽保。推进教育改革发展。圆满完成部省共建教育改革发展试验区建设任务。新建公办幼儿园26所，学前三年教育毛入园率达82.6%，高出全省平均水平5个百分点。改造农村薄弱学校113所，义务教育均衡发展成效明显，武胜县、邻水县顺利通过省级评估验收。普职两类高中办学水平大幅提高，普通高考创历史新高，广安职业技术学院创建省级示范性高职院校通过终期评估。发展卫生计生事业。顺利通过国家卫生城市复审。稳步推进医药卫生体制改革，8家县级公立医院取消药品加成。四川大学华西广安医院挂牌运行。鼓励社会资本合作办医，新审批民营医疗机构20家。全市新农合参合率达99.73%。传染病发病率低于全省平均水平。全面实施“单独两孩”政策，人口自然增长率3.46‰。推动文化体育事业发展。文化惠民工程深入实施，安装直播卫星广播电视“户户通”设备13760户，新建文化院坝34个。国有档案移交进馆和重点档案抢救工作扎实有效。文化遗产保护有序推进，参加第五届中国成都国际非物质文化遗产节，荣获金奖2项。加快发展文化产业，实现产值17亿元。开展第五届“广安文艺奖”评奖工作。成功举行全国第九届残运会暨第六届特奥会圣火采集及首站传递活动，参加省各类锦标赛获金牌23枚。

切实加强生态环境保护。全面贯彻实施新修订的《环境保护法》，重拳惩治环境违法犯罪行为。狠抓大气污染防治，城区空气质量优良率为76.7%。严格淘汰落后产能，依法关闭高污染企业，全市主要污染物化学需氧量、二氧化硫、氨氮、氮氧化物大幅削减，总量减排成效明显。建成乡镇污水处理厂28座，全民水库、西溪河流域水环境治理等重点项目加快建设，全市县级以上城市集中式饮用水源地水质达标率为100%，乡镇集中式饮用水源地水质达标率为93.7%。启动生态保护红线划定工作。深入推进生态市、县创建活动和生态乡镇建设工作。广安区成功创建为国家可持续发展试验区。大力营林造林，新增森林面积9.2万亩，森林覆盖率达到40.2%。

加强和创新社会治理。扎实开展“法律七进”活动，全面完成“六五”普法任务。深化户籍制度改革，“一标三实”基础信息采集综合绩效居全省第二位。“五大民生警务”进一步实施，立体化社会治安防控体系建设深入推进，依法打击各类违法犯罪活动。加强城乡社区网格化管理，畅通群众信访渠道，不断创新大调解“广安模式”，多途径预防和化解社会矛盾。强化安全监管和隐患排查整治，加强专职消防队伍和公共消防设施建设，安全生产形势持续好转。加大食品药品执法监管力度，全市未发生较大以上食品安全事故。推进军地协作配合，健全应急管理体系，成功举办反恐防暴综合实战演练，科学应对“4•4”极端天气灾害，依法处置邻水“5•16”事件，应急处置能力不断提升。国防后备力量建设、民兵预备役、兵员征集、双拥共建和人民防空工作得到加强。

深入推进依法行政。坚持完善政府常务会学法和法律顾问制度，强化规范性文件合法性审查和备案审查。严格执行权力运行“三张清单”制度，市本级和各区市县分别建成行政权力依法规范公开运行平台和电子政务大厅，实现省市县三级互联互通；瞄准权力运行重点领域和关键环节，制定出台或修改完善政府投资项目工程变更审查办法等规定制度，切实把权力关进制度的“笼子”。主动接受市人大及其常委会法律监督、工作监督和市政协民主监督，充分听取民主党派、工商联、无党派人士和人民团体意见，办理人大代表议案建议162件、政协提案256件，办复率和满意率均为100%。深化政府信息公开，大力推行“网络理政”“在线访谈”，开通＂12345＂政府服务热线，有效促进了政民互动。落实全面从严治党、从严治政各项规定及党风廉政建设主体责任，扎实开展“三严三实”专题教育，深化改进政务服务提升行政效能专项活动，政风行风持续转变。强化财政、审计监督和行政监察，深化正风肃纪，支持执纪执法机关查办案件，深入推进预防腐败“广土”工程，政府自身建设不断加强。

　各位代表，2015年各项工作目标任务的完成，标志着“十二五”圆满收官。“十二五”时期，是我市战胜各种困难挑战、奋力推进“两个跨越”极不平凡的五年。这期间，世界经济持续深度调整，我国发展进入新常态，经济下行压力持续加大，资源环境约束趋紧，结构性矛盾突出，经济社会发展形势复杂多变。面对压力和挑战，市委市政府总揽全局、沉着应对，作出一系列新决策新部署，团结带领全市人民攻坚破难、砥砺奋进，完成了“十二五”规划确定的主要目标任务，经济社会发展取得了重大历史性成就，为加快建设经济强市、全面建成小康社会打下了坚实基础。

这五年，是广安战略位势大幅提升，对外影响力显著增强的五年。我们成功争取并全面实施川渝合作示范区、国家级经开区、国家西部承接产业转移示范区等国家战略，为西部地区开展区域合作、承接产业转移提供了经验示范。我们成功取得全国文明城市、国家卫生城市、国家森林城市、全国双拥模范城市等多项桂冠，成功争取国家中小城市综合改革试点城市、国家循环经济示范城市、国家级现代农业示范区、首批国家商标战略实施示范城市、国家住宅产业现代化综合试点城市、国家智慧城市试点城市、部省共建教育改革发展试验区等多项国家政策支持，极大提升了城市美誉度和竞争力。我们成功筹办邓小平同志诞辰110周年纪念活动，广安的知名度和对外影响力得到大幅提升。

这五年，是广安经济发展实现重大跨越，综合实力明显增强的五年。五年来，全市地区生产总值持续保持两位数增长，以年均增长12.2%、分别快于全国全省4.4、1.4个百分点的速度追赶跨越发展，突破1000亿元。人均地区生产总值从2010年的15588元增加到31000元。全社会固定资产投资年均增长27%，突破1000亿元。社会消费品零售总额年均增长15%。全市工业增加值由199.4亿元增加到412亿元。地方一般公共预算收入由21.3亿元增加到56.7亿元，年均增长21.7%。金融机构各项存款余额由638.85亿元增加到1425.33亿元。

这五年，是广安转型升级扎实推进，结构调整取得实质进展的五年。三次产业结构从20.5：48.2：31.3调整为15.8：52.2：32.0，产业创新、融合、成链、集群发展趋势明显；潜力产业和战略性新兴产业产值占工业总产值比重提高8.7个百分点，关闭地方小煤矿39个、淘汰落后产能923万吨。创新驱动战略深入实施，高新技术产业总产值增长18%，获专利1516项，科技对经济增长的贡献率不断提高。绿色发展成效明显，完成中省节能减排目标，主要污染物化学需氧量、二氧化硫、氨氮、氮氧化物分别削减9%、13.4%、5.5%、37%。

这五年，是广安全面深化改革攻坚破难，开放合作卓有成效的五年。创新实施多点多极支撑发展战略，调整行政区划设立前锋区，扩大国家级经开区管辖范围，设立新的市级园区，推动国有资产重组和管理体制改革，形成了多点驱动、竞相发展的新格局。完成新一轮政府机构改革。取消下放92项行政审批事项。农业农村、要素价格、财税、投融资等重点领域改革取得重大进展，改革红利不断释放。创新创业热潮蓬勃兴起。抢抓“一带一路”和长江经济带建设战略机遇，对外开放合作全方位拓展，招商引资取得重大突破，内陆开放型经济加速构建。

这五年，是广安基础设施建设全面加快，城乡面貌显著改善的五年。综合交通枢纽加速构建，高速公路通车里程新增140公里、达360公里；新增铁路通车里程91公里、达153公里，开通至成都动车，广安迈入动车时代。川东北地区进入长江最近的港口--广安港开港运营，常年通航能力达2200万吨。能源保障不断加强。信息化建设取得突破。城市建成区面积由74.2平方公里增加到130.5平方公里，城镇化率提高8.51个百分点。新改建农村公路6100公里，渠系配套520公里，治理水土流失219平方公里，解决了116万农村居民和学生饮水安全问题。森林覆盖率提高5个百分点。新改扩建农民新村聚居点2378个。

这五年，是广安民生社会事业蓬勃发展，人民群众得到更多实惠的五年。民生总投入591亿元，是“十一五”的2.9倍。累计实施省、市民生工程621项，办理惠民实事18.6万件。获全国优秀民生改善典范城市称号。累计新增城镇就业23.18万人。完成39.57万贫困人口脱贫。城乡居民收入年均分别实际增长12.4%、14.4%。城乡居民基本养老保险、医疗保险等社会保障体系基本形成，社会救助水平不断提升。医疗卫生、公共文化服务、各类教育发展水平不断提高。全民健身和竞技体育迈上新台阶。

这五年，是广安依法治市开启新征程，社会政治保持稳定的五年。依法治市纲要深入实施，民主法治建设有序推进。简政放权、行政审批制度改革工作取得新成效。新型社会治理机制建设取得进展。交通安全综合整治攻坚卓有成效，安全生产形势持续好转。防灾减灾体系更加健全，应急救援能力明显提升。立体化社会治安防控体系加速建立，社会治安形势总体平稳。党风廉政建设和反腐败斗争卓有成效，良好政治生态总体形成。

　各位代表，“十二五”期间，特别是过去一年里，中省高度重视广安发展，全市干部群众加倍付出，各方面也给予了我们宝贵支持。在此，我代表市人民政府向全市干部群众，向人大代表、政协委员，向各民主党派、工商联、无党派人士、人民团体和各界人士，向驻广部队、武警官兵和公安民警，向所有支持广安改革发展的同志们、朋友们，表示衷心的感谢！

　我们也清醒认识到，全市经济社会发展中还存在着不少困难和问题，与全国全省的发展差距仍然较大，发展质量和效益还不够高，转方式、调结构、稳增长任务仍然十分艰巨，改革创新有待深化和突破。教育、医疗、文化等公共服务保障能力不足与资源配置不均衡并存，保障和改善民生任务还比较繁重。政府自身建设还需加强，少数政府机关和公务人员还存在“怠政”“懒政”、不依法办事等现象，依法行政和反腐倡廉还需持续用力。对此，我们将高度重视，认真加以解决。

“十三五”发展的主要考虑

　“十三五”时期是全面建成小康社会的决胜阶段。展望未来五年，宏观经济长期向好的基本面不会发生大的改变，中省系列重大战略叠加落地，广安仍将处于重要的战略机遇期。只要我们奋发有为，在新一轮发展中抢占先机，努力把机遇和潜力变为现实，把困难和挑战化为动力，就一定能实现发展的新跃升，确保如期全面建成小康社会。

　根据党的十八届五中全会、省委十届七次全会精神以及市委关于“十三五”规划编制的建议，市政府组织编制了《广安市国民经济和社会发展第十三个五年规划纲要（草案）》，提请本次大会审议后颁布实施。这里，我着重从三个方面简要说明。

关于奋斗目标。对标我市“两个跨越”的宏伟蓝图，《纲要（草案）》从六个方面提出了“十三五”发展目标。一是经济发展质量效益实现大提升。继续保持经济增长快于全国全省的良好态势，不断增强发展平衡性、包容性、可持续性，促进经济更有质量更有效益增长，地区生产总值年均增长9%以上。经济结构战略性调整加快推进，形成一批重要产业集群和产业龙头，经济综合实力和竞争力实现新的提升。二是创新能力实现大提升。创新驱动转型发展见到明显成效，动力结构实现优化升级，自主创新能力显著提升，科技对GDP的贡献率明显提高。三是人民生活品质实现大提升。基本公共服务均等化深入推进，教育科技和文化卫生事业得到更高水平发展，社会保障更加完善，城乡居民收入持续增长，全部消除现行贫困线标准以下贫困人口，实现贫困县、贫困村全部“摘帽”。四是公民素质和社会文明程度实现大提升。全国文明城市创建成果进一步巩固，人民思想道德素质、科学文化素质明显提高，文化产业蓬勃发展，小平故里文化知名度和影响力持续扩大，群众精神文化生活更加丰富。五是生态文明建设实现大提升。国家循环经济示范城市成功创建，环境质量明显改善，主体功能区布局和生态安全屏障基本形成，主要污染物削减量和城市PM10、PM2.5下降指标全面完成省定目标，环境空气质量优良率达到国家规定标准以上。六是改革开放和民主法治建设实现大提升。重要领域和关键环节改革实现重大突破，开放型经济新体制基本形成，依法治市基本方略全面落实，各领域基础性制度体系加快构建，法治广安、平安广安建设深入推进。上述目标既有定性目标，又有定量目标，既有预期性目标，又有约束性目标，既系统考量了“十三五”经济社会发展面临的宏观环境和支撑条件，又充分考虑了全市人民过上美好生活的新期待，体现了战略性、前瞻性、指导性的要求，是对未来五年我市推进“五位一体”建设和改革开放的系统性目标定位，总体上是通过努力能够实现的目标。

关于着力方位。完成好“十三五”发展的目标任务，必须找准主攻方向，以重点突破支撑和保障发展跨越。《纲要（草案）》体现了四个方面攻坚突破的战略考量。一是坚持加快发展，奋力后发赶超。这是基于我市发展实际、长远目标的战略决策。无论是从“人口多、底子薄、欠发达”的基本市情和“发展不足、发展不充分”的主要矛盾考虑，还是从建设经济强市、加快缩小与全省千亿级经济大市差距的更高目标来谋划，都必须保持必要的增长速度。为此，我们在编制《纲要（草案）》时，确立了经济增速高于全国全省的奋进目标。二是坚持转变方式，增强发展动力。这是宏观大势所趋、发展困境所逼。新常态下，经济发展表现出速度变化、结构优化、动力转换三大特点，我市煤炭、水泥等传统产业产能过剩问题突出，新兴产业尚未发展成为接续替代产业，科技创新对经济驱动作用仍不明显，这些都倒逼我们加快转变发展方式，促进新旧动力转换。为此，我们在编制《纲要（草案）》时，将推进改革创新、转变发展方式作为了战略主线，以创新思维和务实举措推进结构性改革，奋力实现转型发展、科学发展。三是坚持问题导向，补齐发展短板。这是突破最大难点、确保决战决胜的关键之举。我市6个区市县均属秦巴山区贫困县，农村贫困面宽、量大、程度深，同时在社会事业发展、生态环境保护、民生保障等方面也存在一些短板，要如期与全国全省同步全面建成小康社会，必须更加突出薄弱环节，加快弥补发展短板。为此，我们在编制《纲要（草案）》时，将弥补短板放在突出位置，作为重要的主攻方向，提出有针对性的政策措施，努力增强发展的协调性和平衡性。四是坚持防控风险，确保行稳致远。我们过去发展沉淀了一些问题，未来几年各方面风险可能积累甚至集中显露，能否有效防控风险，是确保发展行稳致远的关键。为此，我们在编制《纲要（草案）》时，围绕有序化解各类矛盾，坚守安全稳定底线，谋划了多元化防范处置风险体系建设。

关于发展路径。牢固树立并切实贯彻五大发展理念，坚定走符合广安实际的创新、协调、绿色、开放、共享发展之路，这是“十三五”发展的根本遵循。《纲要（草案）》用3个专篇17个章节就此进行了规划。一是坚持创新发展。围绕解决发展动力问题，根据市委《关于全面创新改革驱动转型发展的决定》，分别对实施创新驱动发展战略、全面深化改革、加快产业结构调整等提出了方向和要求。二是坚持协调发展。围绕解决不平衡问题，强调实施多点多极支撑和统筹城乡发展战略，就打造都市核心功能区、发展壮大县域经济、加快发展国家经开区、做大做强经济支撑点以及加快推进新型城镇化、加强基础设施建设、加强文化建设等提出了方向和要求。三是坚持绿色发展。围绕解决人与自然和谐问题，就加快主体功能区建设、加强生态环境保护与建设、推进节能降耗减排、提高防灾减灾能力等提出了方向和要求。四是坚持开放发展。围绕解决内外联动问题，就主动对接“一带一路”和长江经济带发展战略、加强重点区域合作、提升开放型经济发展水平等提出了方向和要求。五是坚持共享发展。围绕解决社会公平正义问题，分别就全力实施精准扶贫、保障和改善民生、创新社会治理体制机制等提出了方向和要求。

　我们坚信，通过全市人民的共同努力，广安发展的道路必将越走越宽广，发展的前景必将越来越美好，“十三五”发展的宏伟蓝图一定能够实现。

2016年工作意见

　2016年，是“十三五”决胜全面小康的开局之年，也是推进结构性改革的攻坚之年。做好政府工作的基本思路是：全面落实党的十八届五中全会、省委十届七次全会和市委四届十次全会部署，以邓小平理论、“三个代表”重要思想、科学发展观为指导，深入贯彻习近平总书记系列重要讲话精神，按照“五位一体”总体布局和“四个全面”战略布局要求，牢固树立和贯彻落实“五大发展理念”，坚持新常态要有新作为，始终保持专注发展转型发展定力，统筹稳增长、调结构、惠民生、防风险，深入实施“三大发展战略”，突出全面创新改革牵引，着力加强结构性改革，加快培育发展新动能，促进发展成果共享，努力实现“十三五”良好开局，推动广安加快迈入转型发展、科学发展快车道。

主要预期目标是：地区生产总值增长9%以上；全社会固定资产投资增长15%以上；社会消费品零售总额增长11.5%以上；地方一般公共预算收入增长10%以上；城乡居民人均可支配收入分别增长8%、10.4%以上；完成省上下达的节能减排任务。

　为实现上述目标，今年重点抓好以下工作：

一、突出投资消费拉动，保持经济平稳较快增长

优化投向稳定投资。主动对接中省重点投资领域，优化投资方向，提高投资效益，集中抓好基础设施、重大产业、生态建设、民生事业等领域投资。扎实开展“项目年”活动，加快推进港前大道等192个重大基础设施项目，广安利尔草铵膦等218个重大产业项目，龙女湖国际康养产业城等156个重大民生项目，官盛新区美好家园等40个重大棚户区改造及房地产项目建设，固定资产投资完成1200亿元以上。激发社会投资活力，探索建立民间投资负面清单管理制度，最大限度缩小审批和核准范围，鼓励社会资本参与公共项目建设，实现社会投资400亿元以上。

顺应需求扩大消费。以新消费引领新供给、打造新动力，引导企业根据消费需求调整供给产品，实现供给与需求相匹配。积极培育文化、旅游、康养等消费新热点、新模式，扩大服务消费、信息消费、绿色消费、农村消费等重点领域，以消费升级带动产业升级。坚持因地制宜、错位竞争、凸显特色，协调推进中央商务区、核心商圈、专业市场建设，组织开展好市场拓展“三大活动”，精心办好名标商品展销会、进出口商品展等活动，提升消费对经济增长的拉动作用。用好线上线下两种资源，提升传统企业电商应用率，鼓励广安名特产品电商化，推广运用互联网、手机等新兴支付方式，力争电商交易额突破100亿元。

调优结构拓展外贸。围绕轻纺、电子等重点产业，着力引进培育加工贸易、服务贸易等生产型出口企业，调优外贸产业结构和产品结构，不断扩大外贸企业规模和总量。提升华蓥省级电子信息专业出口示范基地、邻水省级汽车（摩托车）产品出口基地承载能力，支持前锋区创建省级轻工专业出口基地。深入推进“万企出国门”活动，实现外贸进出口总额8亿美元以上，实际利用外资4000万美元以上。

二、全面深化改革创新，切实增强经济发展新动能

突出抓好结构性改革。按照“去产能、去库存、去杠杆、降成本、补短板”要求，用结构性改革的办法破解发展难题。稳妥化解过剩产能。采取兼并重组、消化淘汰、管控增量等方式，多措并举淘汰落后产能、化解过剩产能；尽可能多兼并重组、少破产清算，实现“僵尸企业”市场出清。帮助企业降低成本。落实中省减税降费政策，加快资源品价格改革，提高生产经营效益，降低企业制度性、外在性、经营性成本。着力化解房地产库存。加大棚改货币化安置力度，满足市民住房需求，促进房地产业健康发展。防范化解金融风险。依法解决涉众性民间借贷，打击非法集资，防范和化解地方政府性债务风险。

持续深化重点领域改革。继续推进农村产权制度改革，依法推动土地经营权有序流转。深化公共交易机制体制改革，搭建国省市统一对接互联互通的电子交易平台。全面推进商事制度改革，积极营造宽松便利和法治化营商环境。深化市属园区管理体制改革，进一步下放市级管理权限，保障园区高效快速发展。大力推进国资国企改革，确保国有资产保值增值。深化国税地税征管体制改革，不断推进税收征管体制和征管能力现代化。理顺政府间财政利益分配关系，科学划分支出责任；深化预算管理改革，完善政府预算体系，健全规范透明的预算制度；转变财政支持发展方式，构建产业发展投资引导基金体系和财政金融互动政策体系。加快金融体制改革，积极推动农村产权抵押融资和小额贷款保证保险试点，全面完成信用联社改制工作，启动建设广安市中小企业金融服务中心，鼓励发展融资租赁、产业基金等新型金融，充分依托多层次资本市场加快实体经济发展。加大投融资体制改革，积极推行PPP等投融资模式，吸引更多社会资本投入公共服务领域。推进现代保险服务业改革发展。加快建立和实施不动产统一登记制度。加快养老保险、医药卫生、教育文化、公务用车等制度改革，全面释放各领域、各行业发展活力。

着力推进创新驱动发展。深入实施创新驱动发展战略，发挥决策咨询暨投资促进专家优势，支持企业联合科研院所、高等院校组建产业技术创新联盟，加快建设中国电子信息产业广安分院、清华大学新能源研究院、西南大学高端产业技术研究院。促进创新资源向企业集聚，加快培育具有较强影响力、拥有自主知识产权的创新型企业，新培育国家高新技术企业4家、省级高新技术产业园区1个、省级特色产业化基地1个，新建省级技术研发中心3个。支持企业转化运用重大科技成果，专利申请量增长15%以上。

全面优化创业创新环境。打造创业创新公共平台，完善创业投资引导机制，设立创新创业引导基金，积极创建国家、省级“双创”示范基地，新建一批大学生创业园、科技孵化园、工程技术中心，为大学生、返乡农民工创业创新提供低成本、便利化、开放式众创空间。实施高新技术企业倍增行动，发挥企业技术创新主体作用，鼓励科研人员创新创业，努力形成想创、会创、能创、齐创的生动局面。新增科技型中小微企业80家以上。

　三、加快产业转型升级，积极构建现代产业体系

以“提质增效”加快工业转型发展。坚定不移实施“工业强市”战略，认真落实“中国制造2025四川行动计划”，深入开展质量对标提升行动，加快新型工业化发展进程，全年实现规上工业增加值增长10%以上。加快发展战略性新兴产业，推动精细化工、高端装备制造、电子信息、新型住宅制造、生物医药、节能环保及新材料等新兴产业突破发展。积极改造提升传统产业，推广运用新技术、新工艺、新设备、新材料，完成技改投资175亿元以上；以市场化机制推进煤炭、水泥企业兼并重组，促其提档升级、做大做强。着力打造工业创新平台，实施制造业创新中心建设、高端装备创新研制等一批重大工程，不断完善创新研发中心、孵化器平台等设施建设。持续提升园区产业集聚能力，推进园区建设特色化、专业化。鼓励支持企业上市发展，利用资本市场做大做强。支持中小微企业“专精特新”发展，全年新增规上企业30户。兑现落实工业稳增长政策措施，切实帮助企业解决市场开拓、要素保障等问题，推动工业经济稳定增长。

以新兴业态引领服务业转型发展。优先发展电子商务、现代物流、现代金融、科技服务、健康养老等新兴先导型服务业，培育发展云服务、e消费、物联网等新业态，积极发展现代会展业和农村服务业，推进服务业与制造业、现代农业互动发展，服务业增加值增长10%以上。鼓励“全企入网、全民触网”，支持广安区建设电子商务孵化园、枣山园区建设省级电子商务产业园、前锋建设川东北电商实训基地、武胜打造众创空间、岳池和广安区发展农村电子商务，网销额增长30%以上。推动广安港、枣山、万善、高兴、前锋重点物流园区及一批重点物流项目建设，物流业增加值增长8%以上。加快推进协兴天星湖、官盛新区康养城建设。提档升级小平故居、华蓥山等旅游景点，加强旅游商品开发，办好四川华蓥山（国际）文化旅游节；借助红色旅游联盟平台，加强省内外交流合作，提升旅游业发展质量，旅游收入增长20%以上。

以“三产融合”助推农业转型发展。以国家现代农业示范区建设为引领，以“三产融合”为路径，以“111”工程建设为载体，加快农业转方式调结构，推动农业转型升级。全面落实粮食安全行政首长责任制，加强耕地保护，稳定发展粮食生产。推进优势、特色产业规模集约发展，新建现代农业产业基地8万亩，新改扩建畜禽标准化规模养殖场50个。大力培育新型农业经营主体，引导大中专毕业生、返乡人员等领办合作社和家庭农场，支持龙头企业采取直接投资、参股经营等方式参与现代农业发展。注重发展农产品精深加工，提升农产品附加值和农业抗御市场风险能力。抓好邓小平故里优质农产品“华蓥山”公用品牌营销，加强“三品一标”认证管理。积极拓展现代农业发展空间，大力发展农村电商、农产品定制等“互联网+”新业态，促进农业与旅游、康养等产业深度融合。做好农业普查工作。

四、着力推进新型城镇化，加快建设和谐宜居城市

提升城市建设管理水平。坚持规划引领，强化刚性约束；深化规划体制改革，调整和完善规划管理权限。推进全国中小城市综合改革试点，积极争取国家海绵城市建设试点，加快国家住宅产业现代化综合试点城市建设。扎实开展“城市基础设施建设年行动”，加快建设城市地下综合管廊，实施万盛片区雨污分流改造及道路综合整治、广前路城区段改造工程，加快神龙山组团路网建设，加大公共停车场、城市慢行系统等基础设施建设力度，不断增强城市承载能力。深入实施“五个统筹”，全面实施居住证制度，加快推进农业转移人口市民化，实现城镇常住人口公共服务全覆盖。改革完善城市管理体制机制，加强城市精细化管理，推动城市管理和城市治理走上法治化轨道。新增城区面积10平方公里以上，城镇化率提高1.6个百分点。

统筹推进区域发展。把县域发展作为城乡统筹发展、决胜全面小康的主战场，夯实多点多极支撑发展底部基础。突出培育县域主导产业。因地制宜分类指导，错位发展重点突破，加快推进县域产业转型升级。加快建设特色产业园区。突出功能分区、特色发展，优化产业空间布局，集中打造特色产业基地，推进广安经开区精细化工及新型住宅产业、枣山园区现代物流商贸、协兴园区文化旅游、华蓥山景区集群集约发展。深化“百镇建设试点行动”，整合用好国家重点镇、省级示范镇优惠政策，加快50个特色集镇建设，全力打造一批特色突出、产业集聚、环境优美、功能完善的特色集镇。大力支持边远乡村发展。切实加大人才、技术、资金倾斜力度，重点支持边远乡村改善发展条件、培育富民产业、增强公共服务能力，统筹实施产村相融、新村扶贫，加快建设370个幸福美丽新村。

着力改善城乡条件。加快重大交通项目建设。G42华蓥站改造、G65邻水南建成通车，完成G350武胜过境段、G210邻水县城西环线、广华快速通道华蓥段、S206同兴至酉溪段等公路建设；加快官盛大桥、广华快速通道广安区段等项目建设；开工建设广安主城区过境高速东环线及渝广高速支线、G212武胜段、S203和S205广安区段、罗渡大桥等9个重大项目；推进高兴至涪陵铁路、兰渝铁路广安支线增建二线、广安机场等项目前期工作；做实镇巴（陕西）至广安高速公路前期工作，力争广安至通江段开工建设；升级改造县乡公路360公里，新改建通村公路800公里，不断提升城乡交通条件。加快重大水利项目建设。快速推进猫儿沟、回龙寺、应家沟、向阳桥、龙滩等中型水库建设，全面启动亭子口灌区前期工作，抓好七一水库、回龙水库、五排水库等中型水库渠系配套及节水改造项目，发挥水利设施作用，改善水资源环境。加快电力、通信、邮政等基础设施建设。推进重点输变电工程建设，实施农村电网升级改造，增强城乡供电保障能力；加快光纤改造、宽带乡村等通讯设施建设，完成585个行政村宽带建设，不断提升城乡信息化水平。

　五、积极扩大对外开放合作，大力提升开放型经济水平

全方位融入国家开放战略。精准对接国家战略布局，主动参与“一带一路”建设，深入实施“2211”三年行动计划，鼓励一批成长型、潜力型企业融入“一带一路”发展，在能源资源、电子信息、装备制造、工程建设等领域，与沿线国家、地区开展合作。主动融入长江经济带和成渝城市群建设，加强与长江上下游节点城市在产业转移、商贸物流、交通运输、生态治理、人文交流等方面合作。

宽领域推进区域合作发展。深化与长三角、珠三角、京津冀、成渝等重点区域合作，组织举办中国（广安）川渝合作示范区投资贸易洽谈会，加快推进广（安）-深（圳）产业园建设。抢抓京津冀一体化产业结构调整机遇，加强与天津滨海新区、天津经济技术开发区、天津港保税区产业合作，力争在金融、电子信息、医药化工、装备制造、旅游开发等方面取得突破。加强与中关村政企互动，建设协同创新共同体，推进高新技术在广安转化。推进与中节能、江苏宜兴环科园战略合作，积极打造川东北节能环保产业基地。务实推进川东北经济区区域合作，共同打造川渝陕甘结合部区域经济中心。依托友城和国际商会，加强与法国、韩国、德国等国家经贸合作。

多层次开展投资促进活动。主动对接京津冀、长三角、珠三角、港澳等区域，举办“感恩小平·投资广安”系列专题投资促进活动，积极参加中外知名企业四川行、川商返乡发展大会、西博会、渝洽会、农博会等重大投资促进活动，开展电子信息、装备制造、生物医药、精细化工等产业专题招商，力争落实一批重大招商项目。抓好已签约项目跟踪落地，提高招商引资项目履约率和资金到位率，帮助企业后续发展。招商引资项目形成固定资产投资520亿元以上。

六、坚决打好脱贫攻坚战，主动顺应群众新期待

　认真落实“六个精准”“六有”要求和“五个一”帮扶制度，大力实施脱贫攻坚工程，加快补齐全面建成小康社会短板。

全面实施脱贫攻坚十个专项方案。坚持精准扶贫、精准脱贫，精细实施十个专项方案年度计划，做到目标、责任、资金、项目、措施、成效“六落实”，在基础设施、产业发展、新村建设、教育与就业、生态建设、医疗卫生计生、文化惠民、社会保障、社会扶贫、财政金融扶贫等十大领域全线发力，实现5万贫困人口脱贫，145个贫困村和3个贫困县“摘帽”。

精准实施脱贫攻坚“五个一批”行动。扶持生产和就业发展一批，稳定解决4.91万人增收问题；大力开发公益性岗位，解决城镇贫困家庭零就业问题；移民搬迁安置一批，帮助0.87万人实现搬迁，摆脱发展限制；低保政策兜底一批，将3.1万人纳入最低生活保障范围；医疗救助扶持一批，对4.06万人实施医疗救助；灾后重建一批，解决678名受灾群众生产生活困难。

深入实施脱贫攻坚五大工程。实施住房扶贫工程，推进农村廉租房建设，解决3500户贫困户住房问题。实施产业扶贫工程，引导扶持贫困村发展特色种养业，新发展特色产业基地3万亩。实施基础扶贫工程，新（改）建农村公路1160公里、供水管网980千米，新建（整治）山平塘600口。实施生态扶贫工程，建立完善生态补偿机制，加快生态恢复，治理石漠化40平方公里，复垦工矿废弃地200公顷。实施智力扶贫工程，优化配置贫困地区教育资源，实施技能培训1.15万人，实现创业就业3500人。

七、加强基本公共服务，切实保障和改善民生

大力实施民生工程。稳步增加民生投入，市级财政不低于65%、新增财力的80%以上投向民生领域。全面完成省定“十项民生工程”和民生大事。集中财力办好市定民生实事，努力增进人民群众福祉。

强化就业和社会保障。落实就业促进政策，城镇新增就业3.5万人，城镇登记失业率控制在4%以内。加大社会保险扩面征缴力度，做好医疗保险异地就医即时结算。整合城乡居民医疗保险制度，实现城乡居民大病保险全覆盖。健全特困人员供养制度，提高低保保障和五保供养标准。完善临时救助制度。促进慈善事业健康发展。

协调发展社会事业。优先发展教育事业。巩固部省共建教育改革发展试验区建设成果，深入推进教育领域综合改革，提高教育水平，促进教育公平。加大以公办幼儿园为主的普惠性幼儿园建设力度，促进学前教育快速发展；加快义务教育学校标准化建设和农村薄弱学校改造；开展普通高中和中职学校学生融合贯通培养试点，推进中高职衔接改革，加快普及高中阶段教育进程；大力发展现代职业教育，支持广安职业技术学院二期项目建设、实施混合所有制改革；支持特殊教育发展。进一步完善公共文化服务体系。加快文化传媒综合体、博物馆等重点项目建设，增强免费开放场馆常态化服务功能；推进公共文化服务机构数字化建设；大力实施文艺精品工程，繁荣文艺创作生产；培育发展文化市场，推动多元文化产业融合发展，加大文化遗产保护力度。加快市体育综合馆和开放式体育场所建设，丰富全民健身活动，发展青少年体育事业，提升竞技体育综合实力。推进“健康广安”建设。深化县级公立医院综合改革；开工建设市人民医院第二住院大楼；加强市中医医院服务能力建设；实施基层医疗卫生服务能力提升工程，积极推进医疗联合体建设、县域医疗卫生一体化管理，大力推行分级诊疗制度。加强新农合资金监管。实施“全面两孩”政策。加强残疾人保障和服务体系建设。

　八、严格保护生态环境，促进人与自然和谐共生

推进“美丽广安”建设。严格划定和控制生态保护红线。加大水土流失综合治理，实施城乡绿化美化工程，加强新一轮退耕还林、天然林资源保护工程建设，营林造林16万亩。抓好生物多样化保护，加强大熊猫野化培训工作，争取设立华蓥山自然保护区。加大湿地保护利用力度，推进武胜县龙女湖省级湿地公园建设，启动建设白云湖国家湿地公园。

落实节能减排行动。积极创建国家循环经济示范市，加快广安经开区国家循环经济示范试点园区改造，启动嘉陵江流域国家生态文明先行示范区建设。强化结构、工程和管理节能减排，推广应用节能减排装备和技术，实施能源和水资源消耗等总量强度双控行动，促进绿色低碳循环发展。单位GDP能耗下降3.5%左右，工业固废综合利用率提高到78%。

加强环境污染防治。贯彻实施环境保护法律法规，加大大气、水、土壤污染防治力度，切实改善环境质量。实施渠江、嘉陵江、御临河、大洪河流域及重点小流域生态环境综合治理，抓好饮用水源保护。深入开展城乡环境治理，加强矿产资源开发利用监管，大力推进“洁净水”行动，达标处理城镇生活污水和城乡垃圾，不断优化生态环境。加强环境执法工作，依法打击环境违法行为。

九、依法推进社会治理，全力维护社会和谐稳定

创新社会治理方式。健全网格化服务管理机制、治安隐患发现机制，提升社会精细化管理水平。完善基层群众自治制度，积极推进社区公共服务综合信息平台建设，增强社区服务功能，实现政府治理和社会调节、居民自治良性互动。健全紧急重大信息发布机制，推进“12345”政府服务热线受理中心建设，及时回应群众关切。建立重点人群人性化、动态化、服务型管理方式，强化重点人员服务管理，做好监督管理、教育矫正和适应性帮扶工作。支持工会、共青团、妇联、工商联等人民团体参与社会治理，形成多元共治局面。

预防化解社会矛盾。扎实开展“七五”普法教育，深入推进法治广安建设。运用法治思维和法治方式化解社会矛盾，维护好群众合理合法利益诉求。健全重大决策社会稳定风险评估机制，落实市、县、乡、村、组五级联合排查联合化解制度，源头防范治理矛盾纠纷。改革信访工作制度，建立畅通有序的诉求表达、心理干预、矛盾调处、权益保障机制，有效防范和化解土地征用、房屋拆迁、民间融资、安全生产等重点行业和领域引发的社会矛盾。深化“大调解”工作，最大限度增加和谐因素。

健全公共安全体系。全面推进平安广安建设，纵深推进“五大民生警务”活动，创新实施“互联网+N”警务模式，加强城乡立体化数字化社会治安防控体系建设，依法严厉打击各类违法犯罪活动，增强人民群众安全感。严格落实企业安全生产主体责任，从严追究生产安全事故责任，加强隐患排查治理和安全预防控制，强化重点行业和领域安全专项治理，坚决遏制重特大安全事故发生。加强食品药品和农产品质量监管，构建食品安全社会共治体系。完善防灾救灾和应急救援体系。继续抓好地质灾害防治工作。加强测绘地理信息市场监管。认真落实民族宗教政策；支持国防和军队改革，加强国防动员和后备力量建设，推动军民融合发展；做好人民防空、国家安全和保密工作，努力维护社会大局稳定。

关于政府自身建设

　各位代表，实现“十三五”宏伟蓝图、完成今年目标任务，需要全市人民共同奋斗，也特别需要各级政府更加奋发有为、积极工作。我们将以改革创新的精神，进一步加强政府自身建设，落实依法治市、全面从严治党各项要求，行使好人民赋予的权利，完成好人民寄予的重托，努力建设人民满意政府。

严格依法行政，推进法治政府建设。认真贯彻《依法治市纲要》。认真执行人大及其常委会的决议、决定，主动接受人民政协民主监督。抓住我市被赋予地方立法权机遇，加快制定在城乡建设与管理、环境保护、历史文化保护等领域的地方政府规章。推进依法科学民主决策，建立健全决策咨询和法律顾问制度，严格落实重大决策终身责任追究制度及责任倒查机制。在条件成熟的领域实行跨部门综合执法，加强和改进执法监督，深化行政执法责任制，严格规范公正文明执法。

坚持务实为民，推进服务政府建设。深入简政放权，继续清理行政管理事项，厘清政府与市场、社会的边界，加快建立“三张清单”。扩大政府购买公共服务范围，更多运用市场化、社会化、信息化方式，不断提高政府公共服务水平。大力推广电子政务。加快政务大厅共享平台建设，全面推行“一窗进出、受办分离”政务服务运行模式。深化政务公开和政府信息公开，着力打造“阳光政府”，让人民群众更好地监督政府。

持续改进作风，推进效能政府建设。巩固提升“三严三实”专题教育成果，持续加强政风行风专项治理，推动作风建设常态化长效化。严格落实厉行节约反对浪费各项制度规定，确保“三公”经费只减不增，进一步提高财政资金使用效益。持续改进文风、会风。推进政府管理创新，运用信息化手段再造政府工作流程，提高行政效能和政府工作透明度。健全抓执行、抓落实工作机制，加强政务督查和效能监督，确保决策部署全面落实。坚决纠正不作为、乱作为等现象，坚决惩治失职、渎职等行为。

强化从严治政，推进廉洁政府建设。认真落实全面从严治党、从严治政各项规定，切实加强政府系统思想政治建设，深入推进机关党建和党风廉政建设，为推动改革发展提供坚强保障。严格落实“一岗双责”，坚持廉政建设与业务工作两手抓、两手硬，发挥行政监察、审计监督作用，突出重点领域、关键环节和重要岗位的监督管理，稳步推进审计全覆盖，切实规范行政权力运行。始终保持惩治腐败高压态势，支持执纪执法机关查办案件，旗帜鲜明反对腐败，继续推进预防腐败“广土”工程及“法纪七进”活动，着力营造风清气正、崇廉尚实、干事创业、遵纪守法的良好氛围。

　各位代表！新常态带来新机遇，新征程更需新作为。让我们紧密团结在以习近平同志为核心的党中央周围，在省委、省政府和市委的坚强领导下，高举中国特色社会主义伟大旗帜，始终保持“逢山开路、遇水架桥、披荆斩棘、勇往直前”的精神状态，紧紧依靠全市人民，再接再厉，开拓创新，扎实工作，努力推动广安加快迈入转型发展、科学发展快车道，为建设经济强市、全面建成小康社会而努力奋斗！
